# Assessment of the Values of Endoglin and Soluble Fms-Like Tyrosine Kinase-1/Placental Growth Factor Ratio Among Women at Risk for Pre-eclampsia: A Cross-Sectional Study

**DOI:** 10.7759/cureus.93723

**Published:** 2025-10-02

**Authors:** Smitha Subramaniam, Pavithra Mourouganandane, Meena T S

**Affiliations:** 1 Obstetrics and Gynaecology, Sree Balaji Medical College and Hospital, Chennai, IND

**Keywords:** high-risk pregnancy, pre-eclampsia, pregnancy-induced hypertension, soluble endoglin, soluble fms-like tyrosine kinase 1

## Abstract

Background: Preeclampsia (PE) is a common complication of pregnancy affecting pregnant women and newborns. PE causes multi-organ disorders and remains one of the main reasons for maternal morbidity and mortality. In addition, PE leads to many complications that can occur in the fetus or newborn.

Objectives: This study aimed to evaluate the serum endoglin and soluble fms-like tyrosine kinase 1 (sFlt-1)/placental growth factor (PlGF) ratio in pregnant women at high risk for PE and normal pregnant women and to assess the sensitivity and specificity of endoglin and SFlt-1/PlGF ratio.

Methodology: A cross-sectional and purposive sampling study was conducted in the Department of Obstetrics and Gynaecology, Sree Balaji Medical College and Hospital, Chrompet, Chennai, a tertiary care hospital in India, from June 2024 to May 2025 for a period of one year. Pregnant women in 24 to 28 weeks of gestation, as calculated by the LMP (last menstrual period) and dating scans, were included in the study. The following were excluded from this study: pregnant women beyond 28 weeks of gestation, pre-eclampsia, hematological disorders, autoimmune disorders, hepatitis, fever, and any systemic infection. Blood samples were collected and used for the analysis of serum endoglin, sFlt-1, PlGF, renal function test (RFT), and liver function test (LFT). Diagnostic accuracy of the biomarkers was assessed by calculating sensitivity, specificity, positive predictive value, negative predictive value, and ROC curve using MedCalc version 15.0 (MedCalc Software, Ostend, Belgium).

Results: In this research, 114 patients were included. Of them, 57 were at high risk for PE, and 57 were considered to be in the control group. The average age of those who took part in the case study was 26.4 ± 4.36 years, while the average age of those who served as controls was 25.73 ± 4.11 years, with ages ranging from 18 to 35. The body mass index (BMI), mean arterial pressure (MAP), and number of primigravida are increased in high-risk groups. Serum endoglin and SfLT-1/PlGF ratio levels were significantly increased in high-risk patients for PE, when compared to the control group.

Conclusion: This study highlights the clinical significance of serum endoglin and sFlt-1/PlGF ratio levels in women at high risk for PE. Elevated serum endoglin and SFlt-1/PlGF ratio levels were found to be strongly associated with women at high risk for PE. Hence, it can be used as a potential biomarker for detecting the high risk for PE at 24 to 28 weeks. This study suggested that using a combination of the above two markers (endoglin and Sflt-1/PGLF ratio) will have a high sensitivity of 100% (43.7-100) and a specificity of 71.9% (58.9-82.3).

## Introduction

Preeclampsia (PE) is a multisystem hypertensive disorder of pregnancy affecting 3-8% of pregnant women and 9-26% of newborns, making it a major contributor to maternal morbidity and mortality. It accounts for nearly 50,000 to 60,000 maternal deaths annually in low- and middle-income nations [1]. While most cases of PE include new-onset hypertension occurring after 20 weeks of pregnancy and often manifesting near term, it is possible for some women to have hypertension along with other symptoms that do not involve proteinuria [2]. Early diagnosis, screening, and treatment of PE can significantly reduce mortality, although its exact cause and mechanisms remain elusive.

In the early stages of pregnancy, PE is characterized by abnormal placentation, defective remodeling of the spiral arteries, and uteroplacental malperfusion, leading to placental hypoxia and maternal endothelial dysfunction. This cascade promotes systemic inflammation, oxidative stress, and altered vascular responses [3]. One of the critical pathogenic mechanisms involves the imbalance between angiogenic and antiangiogenic proteins. Initial evidence showed that levels of placental growth factor (PlGF) decrease while soluble fms-like tyrosine kinase-1 (sFlt-1) levels rise, establishing the utility of the sFlt-1/PlGF ratio as a biomarker of disease progression [4]. A markedly increased sFlt-1/PlGF ratio has been linked to placental abruption, ischemia, vascular lesions, oxidative stress, and maternal endothelial sensitization, all of which contribute to the clinical spectrum of PE [5].

PE also predisposes to severe maternal complications. Hemorrhagic stroke is the most prevalent cerebrovascular event in severe cases, largely driven by uncontrolled hypertension. A study focusing on women with PE and eclampsia reported that 89% of strokes were hemorrhagic, although ischemic stroke risk is also elevated [6]. Renal involvement is another hallmark, with physiological enlargement of the renal pelvis seen in more than 80% of pregnancies due to hormonal changes, further aggravated in PE by hemodynamic strain and proteinuria [7]. Similarly, hepatic microcirculatory abnormalities and hepatocellular necrosis, driven by endothelial failure, explain the liver dysfunction often observed in affected women [8].

The clinical utility of angiogenic biomarkers has advanced PE prediction and diagnosis. The sFlt-1/PlGF ratio is increasingly applied in practice, especially after 20 weeks of gestation, to identify asymptomatic high-risk women. Elevated ratios, particularly when associated with fetal growth restriction, help stratify women likely to experience adverse maternal and perinatal outcomes [9]. Established maternal risk factors such as African or Black ethnicity, history of placental complications, systemic lupus erythematosus, diabetes, obesity, pre-existing renal disease, and abnormal uterine artery Dopplers further amplify susceptibility to PE.

Multiple biomarkers reflecting placental ischemia, endothelial dysfunction, oxidative stress, and systemic inflammation have been investigated. Among these, soluble endoglin, a glycoprotein that inhibits angiogenesis, has emerged as a promising marker. Elevated maternal serum endoglin levels impair vascular remodeling, exacerbate endothelial dysfunction, and are closely correlated with disease onset and severity [10]. While traditional measures such as blood pressure monitoring and proteinuria remain central to diagnosis, their variability and limited predictive accuracy highlight the need for more specific tools. The use of mercury sphygmomanometers, once a diagnostic standard, has raised additional concerns about accuracy and safety in modern practice [11].

In summary, PE arises from complex interactions between abnormal placentation, angiogenic imbalance, and maternal endothelial dysfunction, with wide-ranging systemic consequences. Angiogenic markers such as soluble endoglin and the sFlt-1/PlGF ratio hold significant promise in improving early detection, risk stratification, and clinical management. The present study was undertaken to evaluate serum endoglin and sFlt-1/PlGF ratio in pregnant women at high risk for PE and in normal pregnant women and to assess the sensitivity and specificity of these markers for the prediction of PE.

## Materials and methods

Study setting and ethical approval

The present study was carried out in the Department of Obstetrics and Gynecology at Sree Balaji Medical College and Hospital, Chrompet, Chennai, between June 2024 and May 2025. Ethical approval was obtained from the Institutional Ethics Committee of the college prior to commencement (approval no. 002/SBMCH/IHEC/2023/2070), and written informed consent was obtained from all participants. The study adhered to the principles outlined in the Declaration of Helsinki, and participation was entirely voluntary.

Study design and population

This was a hospital-based cross-sectional study that employed purposive sampling. Pregnant women attending the antenatal clinic were screened for eligibility, and those between 24 and 28 weeks of gestation were invited to participate. Gestational age was calculated using the last menstrual period and confirmed with a dating ultrasound scan. This gestational window was selected because it corresponds to the optimal period for detecting predictive biochemical markers of PE.

Inclusion and exclusion criteria

Eligible participants were women in the second trimester of pregnancy, specifically those between 24 and 28 weeks, who consented to participate and were willing to provide blood samples and undergo Doppler studies. Exclusion criteria included women beyond 28 weeks of gestation, those already diagnosed with PE, and those with hematological disorders, autoimmune diseases, hepatitis, systemic infections, or febrile illness. Pregnant women with pre-existing systemic diseases that could confound the biomarker analysis were also excluded.

Sample size and recruitment

Sample size was calculated for estimating sensitivity with 95% confidence and ±10% precision. Assuming an expected sensitivity of ~96% and a disease prevalence of 14% in the study population [12], the required number of disease-positive subjects was ~16, yielding a total sample size of approximately 114 participants. Accordingly, 114 pregnant women fulfilling the inclusion and exclusion criteria were recruited for the study. All participants underwent detailed clinical evaluation, including demographic details, medical and obstetric history, and family history of hypertensive disorders in pregnancy. General, systemic, and obstetric examinations were performed at the time of recruitment.

Clinical assessment

Blood pressure was recorded on three separate occasions during a single sitting using a calibrated automated sphygmomanometer, with the patient seated comfortably. The first reading was excluded to minimize variability, and the average of the second and third readings was used in the analysis. Mean arterial pressure (MAP) was calculated by applying the standard formula that combines one-third of the systolic and two-thirds of the diastolic blood pressure values. This parameter was considered essential, as elevated MAP is known to be an early predictor of PE.

Blood sample collection and processing

Venous blood sampling was performed between 24 and 28 weeks of gestation. Approximately 5 ml of venous blood was collected under aseptic precautions, with 3 ml transferred to a plain vacutainer and 2 ml to an EDTA tube. The samples were allowed to clot for 30 minutes and then centrifuged at 3000 rpm for 10 minutes. Serum was separated, aliquoted into labeled Eppendorf tubes, and stored at -20°C until the time of analysis.

Biochemical investigations

Serum samples were analyzed for three angiogenic markers: sFlt-1, PlGF, and endoglin. Each marker was quantified using standardized and validated in vitro immunoassays for human serum. These assays were performed according to the manufacturer’s instructions and were designed for quantitative measurement. In addition to biomarker analysis, residual serum was used to perform routine investigations, including renal function tests (RFTs) and liver function tests (LFTs) with the help of an automated clinical chemistry analyzer. Platelet counts were measured in whole blood using an automated hematology analyzer.

Doppler evaluation

All participants underwent uterine artery Doppler velocimetry between 24 and 28 weeks of gestation. Both uterine arteries were assessed, and indices such as resistance index (RI) and pulsatility index (PI) were recorded. The presence or absence of an early diastolic notch was also documented. Doppler values were later correlated with biochemical marker levels to determine their combined role in predicting the development of PE.

Data management

All data, including demographic, clinical, biochemical, and Doppler findings, were recorded and stored in a customized Microsoft Excel 2010 database (Microsoft Corp., USA). To minimize errors, double entry was performed, and random checks were undertaken for accuracy. The database provided the foundation for subsequent statistical analyses.

Statistical analysis

Statistical analysis was performed using MedCalc version 15.0 (MedCalc Software, Ostend, Belgium). Continuous variables were expressed as mean ± standard deviation, while categorical variables were presented as frequencies and percentages. The distribution of continuous data was checked for normality before further analysis. The Kruskal-Wallis test was used for comparisons of continuous variables between groups, while categorical data were analyzed using chi-square tests or Fisher’s exact test, depending on the data distribution. Diagnostic accuracy of the biomarkers was assessed by calculating sensitivity, specificity, positive predictive value, and negative predictive value. Receiver operating characteristic (ROC) curves were generated to identify optimal cutoff values and to evaluate the overall predictive performance of these markers. A p-value of less than 0.05 was considered statistically significant.

## Results

Of the total 114 pregnant women enrolled in the study, 57 were classified as controls and 57 as high risk for PE. The mean age of the participants was similar between groups, with 25.73 ± 4.11 years in the controls and 26.40 ± 4.36 years in the high-risk group. Among controls, 30 (52.6%) were aged <25 years and 27 (47.4%) were between 26 and 35 years, while in the high-risk group, 19 (33.3%) were <25 years and 38 (66.7%) were 26-35 years (p = 0.048). The mean BMI was significantly higher in the high-risk group (25.90 ± 1.40) compared with controls (23.79 ± 2.10; p < 0.001). With regard to parity, primigravidae were more frequent in the control group (29 (50.9%)) compared to the high-risk group (21 (36.8%)), whereas multigravidae predominated in the high-risk group (36 (63.2%)). Pedal edema was not observed in controls but was present in seven (12.3%) high-risk participants (p = 0.006). The mean gestational age was slightly higher in the high-risk group (26.5 ± 0.5 weeks) compared with controls (25.5 ± 0.5 weeks; p < 0.001) (Table 1).

**Table 1:** Basic characteristics of the study participants (n = 114) * Chi-square/Fisher's exact for n(%). Independent sample t-test for mean+SD. p-value <0.05 is statistically significant.

Parameter	Control (n = 57)	High risk for preeclampsia (n = 57)	Test statistic*	p-value
Age (years, mean ± SD)	25.73 ± 4.11	26.40 ± 4.36	t = 0.77	0.44
<25 years, n (%)	30 (52.6%)	19 (33.3%)	χ² = 3.90	0.048
26–35 years, n (%)	27 (47.4%)	38 (66.7%)		
Body mass index (kg/m², mean ± SD)	23.79 ± 2.10	25.90 ± 1.40	t = 5.21	<0.001
Primigravida, n (%)	29 (50.9%)	21 (36.8%)	χ² = 2.14	0.14
Multigravida, n (%)	28 (49.1%)	36 (63.2%)		
Pedal edema, n (%)	0 (0%)	7 (12.3%)	χ² = 7.56	0.006
Gestational age (weeks, mean ± SD)*	25.5 ± 0.5	26.5 ± 0.5	t = 10.0	<0.001

Table 2 shows the high-risk group demonstrated significantly higher blood pressures, with mean systolic (112.28 ± 19.9 mmHg vs. 104.5 ± 17.5 mmHg, p = 0.034), diastolic (74.56 ± 6.9 mmHg vs. 65.08 ± 8.7 mmHg, p < 0.001), and MAP (99.7 ± 8.5 mmHg vs. 91.4 ± 9.0 mmHg, p < 0.001) compared to controls. Albuminuria was observed in five (8.8%) high-risk women, but none in the controls (p = 0.023). The mean platelet count was lower among high-risk participants (1.98 ± 0.5 ×10⁵/µL) compared to controls (2.39 ± 0.4 ×10⁵/µL; p < 0.001). Renal function tests showed elevated mean urea (28.7 ± 3.14 mg/dl vs. 22.3 ± 2.47 mg/dl) and creatinine (0.79 ± 0.16 mg/dl vs. 0.71 ± 0.12 mg/dl) in the high-risk group (p < 0.01 for both). Liver function markers were also significantly higher among high-risk women, including bilirubin (0.89 ± 0.11 mg/dl vs. 0.72 ± 0.09 mg/dl), aspartate transaminase (AST) (31.8 ± 5.2 U/L vs. 24.8 ± 3.6 U/L), alanine transaminase (ALT) (29.5 ± 4.7 U/L vs. 21.5 ± 3.3 U/L), and alkaline phosphatase (ALP) (98.2 ± 9.4 U/L vs. 85.3 ± 7.6 U/L), all with p < 0.001. Total protein was marginally lower in the high-risk group (6.8 ± 0.6 g/dl vs. 7.1 ± 0.8 g/dl; p = 0.032), with serum albumin reduced (3.9 ± 0.5 g/dl vs. 4.3 ± 0.5 g/dl; p < 0.001) and A/G ratio lower (1.34 ± 0.18 vs. 1.53 ± 0.16; p < 0.001), while globulin levels remained comparable (2.9 ± 0.3 g/dl vs. 2.8 ± 0.4 g/dl, p = 0.16).

**Table 2:** Clinical and laboratory parameters of study participants (n = 114) * Chi-square/Fischer's exact for n(%). Independent sample t-test for mean+SD. p-value <0.05 is statistically significant.

Parameter	Control (n = 57)	High risk for preeclampsia (n = 57)	Test statistic*	p-value
Systolic BP (mmHg, mean ± SD)	104.5 ± 17.5	112.28 ± 19.9	t = 2.15	0.034
Diastolic BP (mmHg, mean ± SD)	65.08 ± 8.7	74.56 ± 6.9	t = 6.16	<0.001
MAP (mmHg, mean ± SD)	91.4 ± 9.0	99.7 ± 8.5	t = 4.59	<0.001
Albuminuria, n (%)	0 (0%)	5 (8.8%)	χ² = 5.18	0.023
Platelet count (×10⁵/µL, mean ± SD)**	2.39 ± 0.4	1.98 ± 0.5	t = 4.33	<0.001
Urea (mg/dl, mean ± SD)	22.3 ± 2.47	28.7 ± 3.14	t = 11.0	<0.001
Creatinine (mg/dl, mean ± SD)	0.71 ± 0.12	0.79 ± 0.16	t = 2.87	0.005
Bilirubin (mg/dl, mean ± SD)	0.72 ± 0.09	0.89 ± 0.11	t = 8.05	<0.001
AST (U/L, mean ± SD)	24.8 ± 3.6	31.8 ± 5.2	t = 8.22	<0.001
ALT (U/L, mean ± SD)	21.5 ± 3.3	29.5 ± 4.7	t = 9.67	<0.001
ALP (U/L, mean ± SD)	85.3 ± 7.6	98.2 ± 9.4	t = 8.05	<0.001
Total protein (g/dl, mean ± SD)	7.1 ± 0.8	6.8 ± 0.6	t = 2.17	0.032
Albumin (g/dl, mean ± SD)	4.3 ± 0.5	3.9 ± 0.5	t = 4.27	<0.001
Globulin (g/dl, mean ± SD)	2.8 ± 0.4	2.9 ± 0.3	t = 1.41	0.16
A/G ratio (mean ± SD)	1.53 ± 0.16	1.34 ± 0.18	t = 5.59	<0.001

In the present study, all evaluated biomarkers showed statistically significant differences between the control and high-risk groups. Mean serum endoglin levels were nearly doubled in the high-risk group (15.24 ± 4.55 ng/mL) compared to controls (8.42 ± 2.52 ng/mL; t = 9.891, p < 0.001), suggesting its role as a marker of endothelial dysfunction. Similarly, sFlt-1 concentrations were markedly elevated among high-risk women (6535.02 ± 472.31 pg/mL) relative to controls (2496.93 ± 402.84 pg/mL; t = 49.111, p < 0.001), while PlGF levels were significantly lower in high-risk pregnancies (157.00 ± 12.81 pg/mL) versus controls (315.00 ± 22.87 pg/mL; t = 45.516, p < 0.001), reflecting impaired angiogenesis. Consequently, the sFlt-1/PlGF ratio was substantially higher in the high-risk group (41.92 ± 4.78) compared with controls (7.97 ± 1.46; t = 51.268, p < 0.001), highlighting its strong predictive potential for PE (Table 3).

**Table 3:** Comparison of biomarker levels between control and high-risk groups (n = 114) *Independent sample t-test. p-value <0.05 is statistically significant.

Parameter	Control (mean ± SD)	High risk for preeclampsia (mean ± SD)	t-value*	p-value
Endoglin (ng/mL)	8.423 ± 2.517	15.242 ± 4.555	9.891	<0.001
sFlt-1 (pg/mL)	2496.930 ± 402.844	6535.018 ± 472.310	49.111	<0.001
PlGF (pg/mL)	315.00 ± 22.866	157.00 ± 12.806	45.516	<0.001
sFlt-1/PlGF ratio	7.973 ± 1.455	41.922 ± 4.782	51.268	<0.001

**Figure 1:**
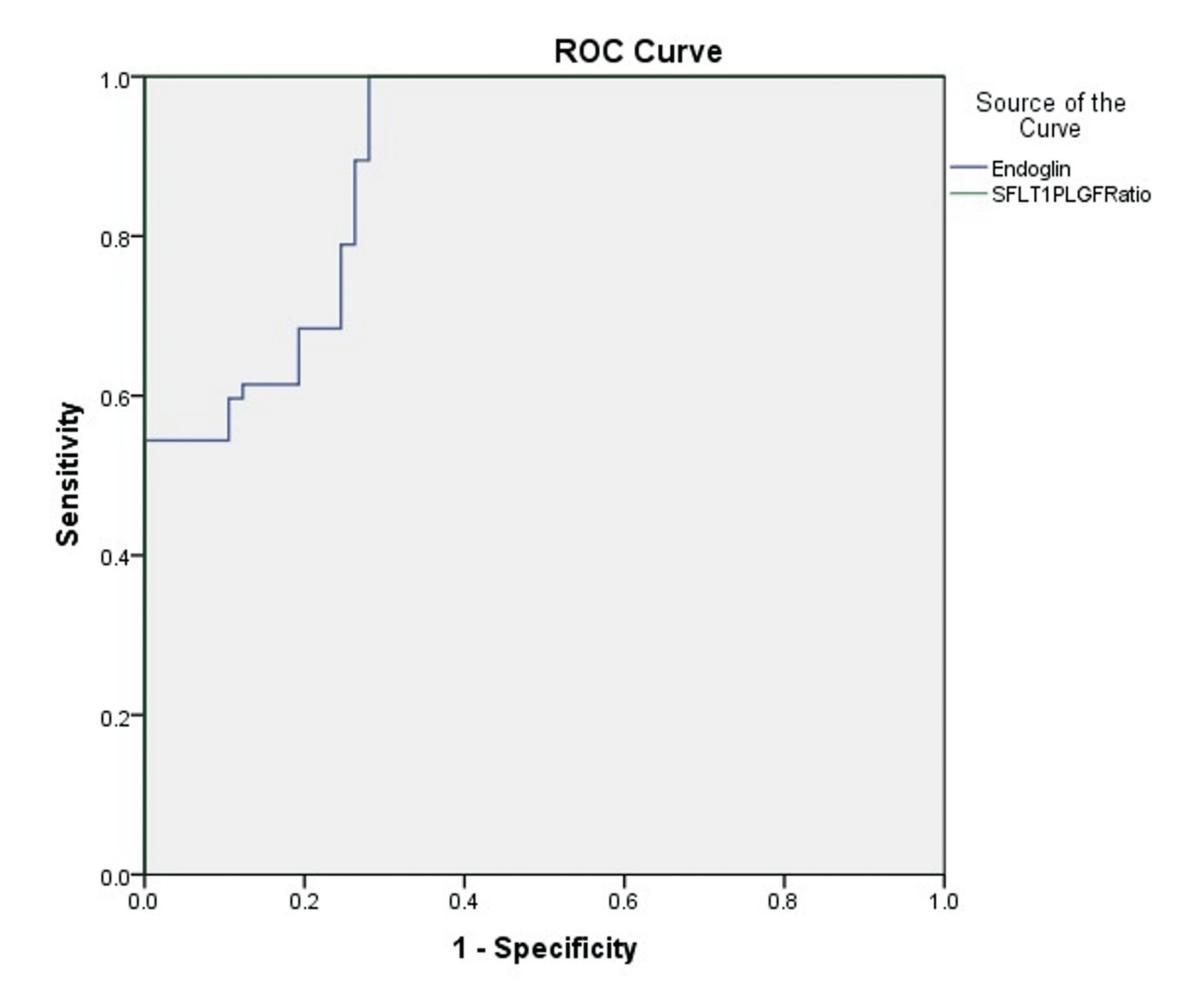
ROC curve for predicting preeclampsia using endoglin and sFlt-1/PlGF ratio ROC: receiver operating characteristic curve

The predictive accuracy analysis demonstrated that both Endoglin and the sFlt-1/PlGF ratio were strong markers for identifying women at high risk of PE. Endoglin showed an AUC of 0.896 (95% CI: 0.840-0.951) with an optimal cut-off value of 9.83 ng/mL, yielding a sensitivity of 100% (43.7-100) and a specificity of 71.9% (58.9-82.3). By contrast, the sFlt-1/PlGF ratio exhibited an AUC of 1.0 (95% CI: 0.75-1) (Figure 1), indicating excellent discriminatory performance, with a cut-off value of 37.93 providing a sensitivity of 78.9% (66.1-88.6) and a specificity of 98.7% (92-99.9). These findings highlight the superior predictive value of the sFlt-1/PlGF ratio, while endoglin remains a highly sensitive marker (Table 4).

**Table 4:** Diagnostic performance of endoglin and sFlt-1/PlGF ratio for preeclampsia AUC: area under curve, PPV: positive predictive value, NPV: negative predictive value

Parameter	Endoglin	sFlt-1/PlGF ratio
AUC (95% CI)	0.896 (0.840–0.951)	1.000 (1.000–1.000)
Cut-off	9.83	37.93
Sensitivity % (95% CI)	100 (43.7–100)	78.9 (66.1–88.6)
Specificity % (95% CI)	71.9 (58.9–82.3)	98.7 (92.0–99.9)
PPV % (95% CI)	61.6 (49.0–73.2)	95.8 (85.7–99.5)
NPV % (95% CI)	100 (52.9–100)	92.8 (83.4–97.5)

## Discussion

PE remains one of the most challenging complications of pregnancy, affecting approximately 3-5% of pregnancies and contributing significantly to maternal and perinatal morbidity and mortality worldwide [13]. Its pathophysiology is characterized by hypertension, endothelial dysfunction, and subsequent multiorgan damage, impacting the placenta, brain, liver, kidneys, and blood vessels. The disease spectrum ranges from maternal complications, such as renal failure, seizures, stroke, and hepatic rupture, to fetal complications, including growth restriction, preterm birth, and perinatal death. Delivery remains the only definitive treatment, underscoring the urgent need for early prediction and risk stratification tools. In this study, we evaluated maternal characteristics and angiogenic markers, i.e., serum endoglin, sFlt-1, PlGF, and the sFlt-1/PlGF ratio, in predicting PE among Indian women. Our findings demonstrated significant differences in maternal age, BMI, MAP, and biochemical profiles between high-risk women and controls, supporting the integration of clinical and biomarker data for early risk assessment.

Advanced maternal age has long been linked to an increased risk of hypertensive disorders in pregnancy. Our study showed a statistically significant difference in maternal age distribution, with many high-risk cases clustering between 26 and 30 years. This finding resonates with the findings of Rymer-Haskel et al. [14], who demonstrated that women older than 35 years with PE were more prone to acute kidney injury compared to their younger counterparts. The vulnerability of older mothers likely reflects reduced renal reserve and impaired vascular adaptability [15]. Together, these observations highlight maternal age as a relevant component of risk scoring models in PE. Obesity and overweight have emerged globally as prominent risk factors for hypertensive disorders in pregnancy. In our study, women at high risk for PE had a significantly higher mean BMI (25.9 vs. 23.7 in controls). The World Health Organization has documented a rising trend in the mean BMI among women of reproductive age, increasing from 22 kg/m² in 1975 to 24.6 kg/m² in 2016 [16]. Elevated BMI not only predisposes to hypertension but also aggravates systemic inflammation and endothelial dysfunction, reinforcing the role of obesity as a modifiable target in antenatal care.

Blood pressure indices, particularly MAP, also showed strong associations in our cohort. High-risk women had significantly higher systolic, diastolic, and MAPs than controls, with MAP >85 mmHg correlating with elevated gestosis scores. This finding aligns with evidence that MAP is a reliable early hemodynamic marker of defective placentation and abnormal trophoblast invasion [17]. Monitoring MAP at routine visits may therefore be a simple and cost-effective predictor in resource-limited settings. Parity has also been implicated in the risk of PE. In our study, 60.5% of women were primigravida, consistent with Wathen et al., who reported a higher prevalence of PE in first pregnancies [18]. Immunological maladaptation and reduced maternal tolerance to paternal antigens in primigravid women are thought to underlie this association. While multiparity is protective in many cases, recurrence of hypertensive disorders in subsequent pregnancies remains a concern.

Clinical markers such as pedal edema and albuminuria were more common in the high-risk group. Pedal edema, although nonspecific, may indicate capillary leak and endothelial dysfunction in PE [19]. Similarly, albuminuria reflects altered renal hemodynamics and glomerular hyperfiltration. Our findings agree with studies that demonstrate increased albumin excretion during pregnancy as both a physiological adaptation and a pathological marker of hypertensive disorders [19,20]. Hematological and biochemical changes were also evident. While platelet counts did not differ significantly, likely due to the predominance of moderate PE in our cohort, subtle reductions were noted in high-risk women. This supports prior evidence that thrombocytopenia becomes more prominent in severe or late-stage disease [21]. In addition, liver function tests showed elevated SGOT, SGPT, and ALP in the high-risk group, coupled with lower serum protein and albumin levels. These findings are consistent with reports that hypoalbuminemia and elevated liver enzymes reflect hepatic microcirculatory dysfunction and worsening maternal outcomes in PE [22,23].

At the biomarker level, our study confirmed significantly elevated serum endoglin and sFlt-1, along with reduced PlGF, in high-risk women compared to controls. These results mirror the pathophysiological model of angiogenic imbalance in PE, wherein anti-angiogenic factors antagonize vascular endothelial growth factor (VEGF) and PlGF, impairing placental perfusion [24]. Venkatesha et al. described the role of soluble endoglin in blocking TGF-β signaling, leading to vasoconstriction, hypertension, and proteinuria [25]. Similarly, sFlt-1, secreted primarily by the placenta, binds circulating PlGF and VEGF, causing endothelial dysfunction [26]. Elevated sFlt-1 levels have been observed even before the clinical onset of hypertension or proteinuria, supporting its predictive value [27]. In our analysis, endoglin achieved excellent sensitivity with excellent negative predictive value, making it useful for ruling out PE. Conversely, the sFlt-1/PlGF ratio demonstrated near-perfect specificity and high positive predictive value, making it a robust confirmatory test. Together, these biomarkers offer complementary diagnostic utility, with implications for individualized risk stratification in clinical practice. The sFlt-1/PlGF ratio demonstrated an AUC of 1.0, indicating perfect discrimination between diseased and non-diseased groups. While this appears ideal, such a result should be interpreted with caution, as perfect diagnostic accuracy is biologically improbable in real-world clinical practice, with wider confidence interval values, which could explain why the actual value is still not 1; it varies from 0.7 to 1.

This study has certain limitations that warrant consideration. First, it was conducted at a single center with a modest sample size, which may limit the external validity and generalizability of the findings. The reported cut-off values for biomarkers are therefore highly specific to this study population and cannot be extrapolated to broader clinical settings without validation in larger, multicenter cohorts. Second, the cross-sectional design restricted our ability to assess longitudinal changes in biomarker levels across gestation, which could have provided valuable insights into temporal trends and predictive dynamics. Third, while three angiogenic biomarkers were analyzed, the inclusion of additional markers or integration with Doppler indices might have enhanced diagnostic accuracy. Finally, potential measurement error and assay variability were not systematically addressed, which may influence the reproducibility of results

Despite these limitations, the findings underscore the value of integrating maternal clinical factors with angiogenic biomarkers to enhance early prediction of PE. Given the simplicity of measuring MAP and BMI alongside serum assays, this approach can be applied in both tertiary centers and resource-constrained settings. The complementary role of endoglin and the sFlt-1/PlGF ratio offers clinicians a practical algorithm, using endoglin to exclude and sFlt-1/PlGF to confirm high-risk cases. The adoption of such strategies in routine antenatal care may allow timely initiation of preventive measures, such as aspirin prophylaxis and closer surveillance, ultimately improving maternal and neonatal outcomes.

## Conclusions

This study suggests that combining maternal characteristics with angiogenic biomarkers may enhance the early prediction of PE. Clinical variables such as maternal age, BMI, and mean arterial pressure, when analyzed alongside biomarkers including endoglin, sFlt-1, and PlGF, improved risk stratification in our cohort. Endoglin demonstrated excellent sensitivity and negative predictive value, supporting its potential utility as a rule-out marker, whereas the sFlt-1/PlGF ratio showed high specificity, consistent with a confirmatory role. These findings align with the biological mechanisms of PE, namely, endothelial dysfunction and angiogenic imbalance. However, given the single-center, modest sample size, and cross-sectional design, the reported cut-off values and diagnostic performance should be interpreted with caution. Larger, multicenter, longitudinal studies are needed to validate these results and determine their applicability in broader clinical practice.
